# Education of the primary health care staff based on acceptance and commitment therapy is associated with reduced sick leave in a prospective controlled trial

**DOI:** 10.1186/s12875-021-01526-5

**Published:** 2021-09-08

**Authors:** Åsa Kadowaki, Anna-Karin Alvunger, Hanna Israelsson Larsen, Anna Persdotter, Marta Stelmach Zak, Peter Johansson, Fredrik H. Nystrom

**Affiliations:** Department of Health, Medicine and Caring Sciences, Faculty of Medicine and Health Sciences, Linköping University, Linköping, Sweden

**Keywords:** Acceptance and commitment therapy, Mental health, Musculoskeletal pain, Treatment efficacy

## Abstract

**Background:**

The use of cognitive behavioral therapy (CBT) to cope with mental distress and pain issues has helped many patients in primary health care in Sweden. However, the effects of CBT to reduce sick leave has not been equally convincing. Acceptance and Commitment Therapy (ACT) is an evolution of traditional CBT and we aimed to study if education based on ACT of the staff rather than the patients could reduce sick leave in primary health care.

**Methods:**

This was a prospective trial in 6 primary health care centers in Kalmar (total amount of registered subjects of 28,930) in which the staff (physicians, nurses and therapists) received group-based education according to ACT during 2018 and 2019. The effects were compared with 5 similarly sized control health care centers in the neighboring Region of Jönköping in which no such education took place. The main aim was to study changes in sick leave in the 6 primary care centers of Kalmar and to keep track of more general trends by studying sick leave also in Jönköping, letting sick leave in the year 2017 to be the reference period for both areas.

**Results:**

The staff at the health care centers in Kalmar reported to having attended a mean of 5.2 ± 2 educational ACT-sessions with psychiatrist Kadowaki in Kalmar. Sick leave for ICD-10 F43 (reaction to severe stress and related adjustment-disorders) was reduced from a mean value of 28.7 ± 9.1ongoing sick leaves/month in 2017 to 22.6 ± 7.0 sick leaves/month in 2018 (-21%, p = 0.033) and to 18.1 ± 10 sick leaves/month in 2019 (-37%, p = 0.038). The corresponding sick leave for any diagnosis (total sick leave) was reduced from 132 ± 39 sick leaves/month in 2017 to 118 ± 38 sick leaves/month in 2018 (-11%, p = 0.056) and to 102 ± 37 sick leaves/month in 2019 (-21%, p = 0.021). The corresponding sick leave comparisons in the control health centers did not show any significant changes (all p-values ≥ 0.24).

**Conclusions:**

Total monthly mean sick leave was reduced 21% in the health care centers in Kalmar during the second year of the educational ACT intervention of the staff while it was unchanged in Jönköping. This suggests a significant effect to induce a reduction in long-term sick leave for patients in primary health care in which the staff received education according to ACT. The results of this trial could serve as a basis for a randomized trial in order to ascertain causality.

**Trial registration:**

Pre-registration November 9, 2018 on ClinicalTrials.gov with number NCT03737019.

**Supplementary Information:**

The online version contains supplementary material available at 10.1186/s12875-021-01526-5.

## Introduction

The quantity of patients with psychosocial and mental problems such as depression, anxiety, and insomnia are increasing in Swedish primary care [1]. Approximately 45% of all ongoing sick leave covered by the Swedish Social Insurance Agency is due to psychiatric diseases according to official statistics. Since a majority of patients with depression or anxiety disorders in Sweden are treated in primary healthcare according to statistics from Swedish National Board of Health and Welfare, the assessments by general practitioners (GP) are of the utmost importance when a prescription for sick leave is called for by the patient. However, this task is considered both burdensome and stressful by Swedish GP [2]. Psychosocial and mental health issues are often manifested as musculoskeletal diseases [3, 4] and when musculoskeletal pain is added to this sick leave equation, the sum represents the largest group for sickness absence [5] in Sweden. Several methods to address and diminish the suffering of the patients (and great societal cost) regarding musculoskeletal and psychosocial problems have been tested. However, multidisciplinary team-based interventions aimed to help the individual patients have so far not become routine, for example, since the effects are controversial. Some trials found minute effects [6, 7] and in one case even a trend to worsen the problems [8]. In contrast, the use of cognitive behavioral therapy (CBT) has accomplished increased well-being of individual patients presenting with psychosocial problems and psychologists now systematically apply CBT in many primary health care centers in Sweden [5, 6, 9]. However, when focusing on the effects of CBT treatment of patients to reduce sick leave, the trials give a more controversial impression. Whereas a small number of studies have demonstrated a reduction [10–12], other trials have reported no or small effects [13, 14]. This is also reflected in the general perception about sick leave for such problem areas, in which both the rationale and effects of CBT have been questioned by GP [2].

Acceptance and Commitment Therapy (ACT) is an evolution of the traditional CBT and it incorporates “a behavior-analytically-based psychotherapy approach that attempts to undermine emotional avoidance and increase the capacity for behavior change” [15]. The aim is to help the subject to develop a psychological flexibility and adaptability to the present disabilities and to achieve a stronger sense of self-acceptance. Another component of ACT is to support the patients to commit to actions that can facilitate the ability to embrace the challenges of life in a productive manner. Since ACT implies that you are trained to commit to face problems head-on rather than to avoid them, education according to ACT in psychiatric and pain related concerns could potentially reduce the requirement for sick leave. Indeed, several earlier trials have successfully employed ACT in clinical trials [16–20] some of which reduced sick leave prescription [16, 18]. However, to the best of our knowledge, all previous studies of ACT to reduce sick leave in primary care were designed to treat the individual patients. In contrast, we aimed to test a new strategy that potentially could allow coverage of larger amounts of patients by employment of group-based ACT education of the medical staff, rather than the patients, in primary health care. Our hypothesis was that we could affect the treatment mindsets and attitudes of the physicians, nurses, and therapists by such education to achieve a more disability-accepting approach towards the symptoms of the patient, and that this would generate a tolerance for the disorders that helped in an attempt to face the problem that was the cause of the visit to the primary care center. We hypothesized that this would be beneficial in the recovery process and hence to reduce sick leave prescription. With these aims we performed a prospective trial in 6 primary health care centers in Kalmar and recruited equally sized control centers in the neighboring Region of Jönköping in which no ACT education was given during the study period. Our main outcome was to analyze the yearly change in sick leave prescription in both areas as reported to official registries.

## Materials and Methods

The personnel involved in clinical care related to sick leave certificates, i.e. physicians, nurses, physical- and occupational therapists at the 6 primary health care centers Emmaboda, Esplanaden, Högsby, Ljungbyholm, Stora Trädgårdsgatan and Färjestaden in Region Kalmar were given ACT based education that was led and organized by psychiatrist Åsa Kadowaki in cooperation with psychologist Mats Dahlin. This general large project to reduce sick leave started in the entire Kalmar Region January 2018 and continued through December of 2019. The personnel of the health care centers could attend 2 + 1 days educations, therapy sessions and/or individual lectures and they could also be guided in the clinical care of individual patient cases aided by ACT at local meetings that started late in 2018 in the 6 participating centers. There were also educational activities with a specialist in pain treatment that started late in 2018 in the city of Kalmar and in the early spring of 2019 in Esplanaden and Stora Trädgårdsgatan (i.e. located in the city of Västervik being a part of Region Kalmar). A more detailed description of the lecture scenarios at each center is given in the supporting documentation file.

We used 6 primary health care centers in the neighboring Region of Jönköping as control centers: Eksjö, Mariannelund, Gnosjö, Gränna, Vaggeryd and Vetlanda in which no ACT based education took place, Region Jönköping is a separate health care organization. In the analysis of this report, data from Eksjö and Mariannelund were counted as one center, since Mariannelund is a subsidiary to Eksjö Health Care Center.

The participants at the health care centers in Kalmar were offered lectures and discussions on repeated occasions and for logistical reasons, at most about half of the work force could participate on each occasion, as determined ad hoc by the head of each center. The educations were mainly aimed towards physicians, nurses, physical therapists, rehabilitation coordinators and occupational therapists but the local program for education was adapted to each center as they differed in size and exact organization. However, any employee that worked directly or indirectly with sick leave certificates being > 18 years old could participate in the educational activities and hence be asked to fill out the questionnaires in this trial. The directors of the 6 primary health care centers in Kalmar approved education during regular working hours. The ACT based group sessions started in the late autumn of 2018 in the 6 health care centers and the participants were asked to fill out questionnaires, anonymously, before the education and after one year for assessment of the well-being of the staff. The results of these questionnaire-data will be reported in a separate analysis, in this report we only used the data on participation in educational activities. The second set of questionnaires delivered after one year also included questions about the extent of attended educational activities. Since the main project to reduce sick leave in Region Kalmar in primary care started in January of 2018, some staff could potentially also attend meetings conveying ACT already from the start of 2018 at neighboring health care centers that were not part of this trial. It is also possible that some staff moved from one health care center to another during the study period. To avoid effects of such spill-over of educational effects from other Health care centers to affect the results, we regarded the complete year of 2018 as being an intervention period in the statistical analyses.

Data on sick leave corresponding to the patients belonging to the health care centers were acquired from the company Inera (www.Inera.se, Tjärhovsgatan, Stockholm, Sweden) which routinely provides such information to the Regions of both Kalmar and Jönköping. The data were based on the number of subjects with any (or specified according to different diagnoses) prescribed ongoing sick leave during each month at each of the health care centers of this trial.

Our primary aims were to address sick leave diagnoses under ICD-10 F43, i.e. reaction to severe stress and related adjustment-disorders, and to study sick leave for any diagnosis, i.e. total sick leave. Secondarily we also addressed any psychiatric disease under ICD F00-99 (denoted F99) and musculoskeletal health issues belonging to ICD M00-99 (denoted M99).

### Statistical analyses

Statistical estimates were calculated using IBM SPSS Amos 27 software (IBM Corporation, Somers, New York, USA). Comparisons between groups were performed with Independent-Samples T test based on mean ongoing monthly sick leave during the years 2017, 2018 and 2019. Students T-test were primarily used for analyzing change within the health care centers since data were based on 12 mean values from 12 months. Non parametric tests within group were also performed by Wilcoxon signed-rank test, as given in the text. Since we could not find any study in which education according to ACT was given to the clinicians in primary care, rather than to the actual treated patients, we did no power calculation. In this sense, this trial should be viewed as a pilot trial. Data are presented as mean values with standard deviations, a two sided p < 0.05 was considered statistically significant.

## Results

There were 28,930 subjects registered in total in 2017 in the 6 health centers in Jönköping, as shown in Table 1. The corresponding figure for Kalmar was 28,255 subjects. On average the 90 respondents on the queries after 12 months reported having participated in a mean of 5.2 ± 2 educational sessions with doctor Kadowaki. However, many respondents to the questionnaire did not specifically respond to the semi-quantitative question on amount of attended educational sessions (20% of records had lack of the data) and we received no filled-out questionnaires at all from the health care center Högsby for logistical reasons. About 15% of the staff in the 6 participating primary care centers in Region Kalmar reported to having attended the dedicated 3-day course led by psychologist Dahlin.

**Table 1:** Effect on ACT based CBT of the staff on prescription of sick leave certificates during the years 2018 and 2019 in primary health care centers in Region Kalmar and Jönköping. The intervention started January 2018 in Kalmar and the year 2017 served as control in both Regions. The mean number of subjects with sick leave certificates was calculated from monthly records of ongoing sick leave

**Primary health care center**	Registered subjects 18–65 years of age	Sick leave F43 2017	Sick leave F43 2018	P-value between 2018–2019	Sick leave F43 2019	P-value between 2017–2019	Total sick leave 2017	Total sick leave 2018	P-value between 2017–2018	Total sick leave 2019	P-value between 2017–2019
**Jönköping**											
Eksjö and Mariannelund	7311	97.0	106.8		120.7		326.5	327.4		341.8	
Gnosjö	5817	25.8	30.8		36.2		171.8	194.3		185.1	
Gränna	2971	24.9	26.2		26.6		88.2	85.7		77.3	
Vaggeryd	3982	27.8	25.1		26.2		140.2	141.6		129.9	
Vetlanda	5663	54.1	50.1		52.5		173.1	175.0		172.8	
Sick leave (mean subjects/month)		45.9 ± 31	47.8 ± 34	0.50 0.50^a^	52.4 ± 40	0.25 0.28^a^	179.8 ± 89	184.8 ± 90	0.34 0.34^a^	181.4 ± 99	0.79 0.69^a^
**Kalmar**											
Emmaboda	4938	21.7	18.8		18.5		90.2	97.7		92.2	
Esplanaden	6237	42.8	31.8		23.5		192.5	183.2		155.2	
Högsby	3048	16.7	14.6		7.5		87.1	69.2		50.5	
Ljungbyholm	3612	32.2	18.7		15.1		142.8	111.7		89	
Stora Trädgårdsgatan	5284	27.5	21.0		9.2		138.9	116.3		92.1	
Färjestaden	5136	31.5	30.6		34.8		139.3	131.6		131.6	
Sick leave (mean subjects/month)		28.7 ± 9.1	22.6 ± 7.0	0.033 0.028^a^	18.1 ± 10	0.038 0.075^a^	131.8 ± 39	118.3 ± 38	0.056 0.046^a^	101.8 ± 37	0.021 0.046^a^

*Abbreviations*: *ACT* Acceptance and Commitment Therapy, *CBT* Cognitive Behavioral Therapy; F43, diagnosis of reaction to severe stress and related adjustment-disorders

^a^ Calculated with Wilcoxon signed-rank test

**Fig. 1:**
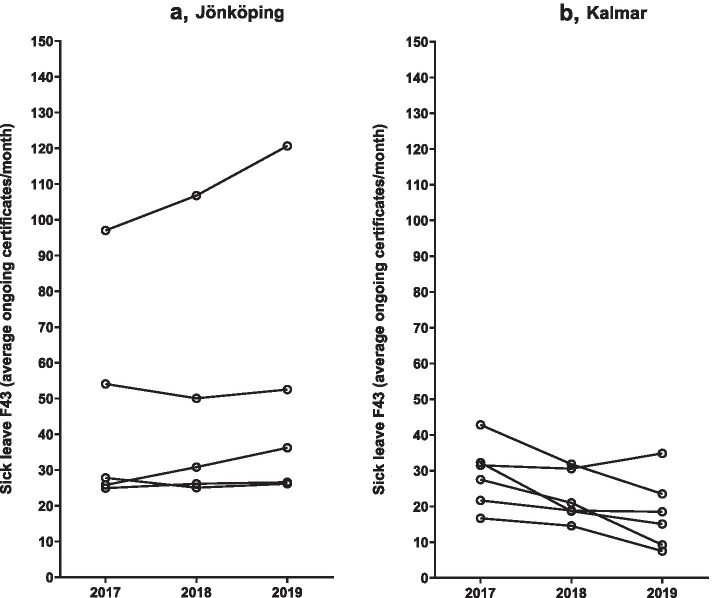
Effect of ACT-based CBT on prescription of sick leave certificates in primary health care centers during the years 2018 and 2019 in Kalmar (6 health care centers, intervention area) and in Jönköping (5 health care centers, control area). The intervention started January 2018 and the year 2017 served as control. The yearly number of sick leave certificates were calculated from mean ongoing sick leaves each month. **a** (Jönköping) and **b** (Kalmar) show sick leave for ICD-10 F43 (reaction to severe stress and related adjustment-disorders)

**Fig. 2:**
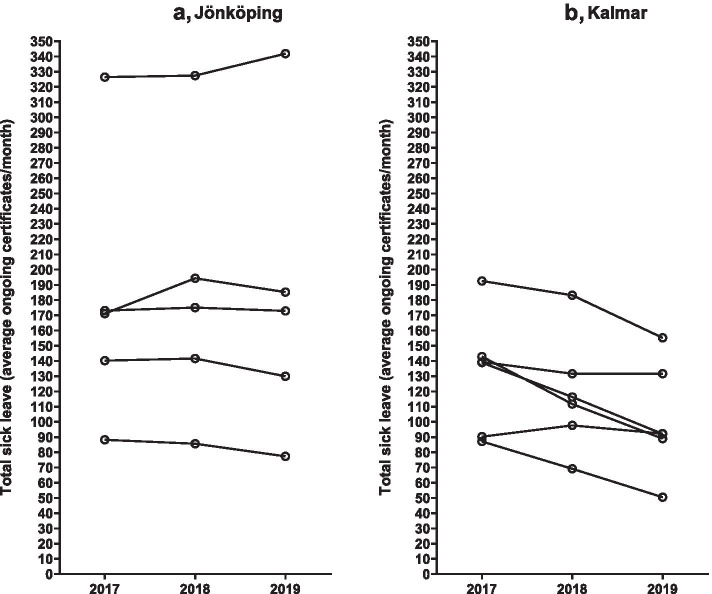
Effect of ACT-based CBT on prescription of sick leave certificates in primary health care centers during the years 2018 and 2019 in Kalmar (6 health care centers, intervention area) and in Jönköping (5 health care centers, control area). The intervention started January 2018 and the year 2017 served as control. The yearly number of sick leave certificates were calculated from mean ongoing sick leaves each month. **a** (Jönköping) and **b** (Kalmar) show mean ongoing sick leave prescriptions for any diagnosis based on monthly records

As shown in Table 1 and in Fig. 1a and b average monthly sick leave based on the ICD-10 diagnosis F43, i.e., reaction to severe stress and related adjustment-disorders, was reduced in the health care centers in Kalmar and remained unaltered in the 5 control centers during the study period. In Kalmar, the sick leave for ICD-10 F43 was reduced from a mean value of 28.7 ± 9.1 sick leaves/month in 2017 to 22.6 ± 7.0 sick leaves/month in 2018 (-21%, p = 0.033) and to 18.1 ± 10 sick leaves/month in 2019 (-37%, p = 0.038). The corresponding sick leave for any diagnosis (total sick leave) was reduced from 132 ± 39 sick leaves/month in 2017 to 118 ± 38 sick leaves/month in 2018 (-11%, p = 0.056) and to 102 ± 37 sick leaves/month in 2019 (-21%, p = 0.021). The corresponding sick leave figures remained unchanged in the control health centers in Jönköping (all p-values = 0.24 or higher. See Table 1 and Fig. 2a and b).

There were also statistically significant differences between changes in mean monthly sick leave figures between 2017 and 2019 for Jönköping and Kalmar (change in F43 in Kalmar: – 10.6 ± 9.3 sick leaves/month, in Jönköping: 6.50 ± 11 sick leaves/month, p = 0.023, change in total sick leave in Kalmar: -30.0 ± 22 sick leaves/month, in Jönköping: 1.60 ± 13 sick leaves/month, p = 0.018 by unpaired T-test).

In an additional calculation we gathered results on the ongoing long-term sick leave certificates/month focusing on those that prescribed a leave lasting 3–12 months. Data were calculated based on January of 2017 (first control month of the study) and compared with those of the end of the period, i.e. December of 2019 in the health care centers of Kalmar. We confirmed a reduction from 36.7 ± 15 long-term sick leaves/month to 20.7 ± 8.0 total sick leaves/month, p = 0.020 for any diagnosis in this analysis.

All the comparisons of sick leave were also tested with Wilcoxon rank sum tests and this rendered minor differences compared with parametric analyses (see Tables 1 and 2). The comparisons of sick leave in Kalmar regarding F43 was no longer statistically significant when comparing 2017 with 2019 in this non-parametric calculation (new p = 0.075) while total sick leave comparing 2017 with 2018 in Kalmar now had a p-value of 0.046 (being 0.056 with the T-test). There were no statistically significant changes in Jönköping in any of these non-parametrical comparisons.

**Table 2:** Effect on ACT based CBT of the staff on prescription of sick leave certificates during the years 2018 and 2019 in primary health care in health care centers in Region Kalmar and Jönköping. The intervention started January 2018 in Kalmar and the year 2017 served as control in both Regions. The mean number of prescribed sick leave was calculated from monthly records of ongoing sick leave

**Primary health care center**	Sick leave F99 2017	Sick leave F99 2018	P-value between 2017–2018	Sick leave F99 2019	P-value between 2017–2019	Sick leave M99 2017	Sick leave M99 2018	P-value between 2017–2018	Sick leave M99 2019	P-value between 2017–2019
**Jönköping**										
Eksjö and Mariannelund	168.83	178.50		193.83		74.00	70.83		65.17	
Gnosjö	56.75	67.33		71.08		60.75	65.17		61.75	
Gränna	52.42	45.75		43.58		16.83	19.00		16.75	
Vaggeryd	54.92	44.83		54.92		42.92	52.58		44.67	
Vetlanda	84.83	85.92		87.33		50.42	49.67		52.67	
Sick leave (mean subjects/month)	45.9 ± 31	47.8 ± 34	0.50 0.69^a^	52.4 ± 40	0.33 0.27^a^	49.0 ± 21	51.4 ± 20	0.33 0.34^a^	48.2 ± 19	0.72 0.69^a^
**Kalmar**										
Emmaboda	29.67	36.75		36.25		23.58	26.50		25.92	
Esplanaden	84.25	70.67		53.25		46.17	46.33		43.75	
Högsby	23.17	20.17		11.42		31.50	22.58		16.58	
Ljungbyholm	63.50	44.00		31.83		35.83	25.75		27.67	
Stora Trädgårdsgatan	54.92	44.00		26.08		41.08	34.83		26.50	
Färjestaden	60.25	55.83		59.33		30.25	29.42		30.67	
Sick leave (mean subjects/month)	52.6 ± 23	45.2 ± 17	0.11 0.12^a^	36.4 ± 18	0.062 0.075^a^	34.7 ± 8.1	30.9 ± 8.7	0.14 0.17^a^	28.5 ± 8.8	0.098 0.12^a^

*Abbreviations: ACT* Acceptance and Commitment Therapy, *CBT* Cognitive Behavioral Therapy; F99, diagnosis of any psychiatric disease; M99 diagnosis of any musculoskeletal disorder

^a^ Calculated with Wilcoxon signed-rank test

Table 2 shows analyses of all diagnoses under F99 (any psychiatric disease) and M99 (musculoskeletal disorder). We found statistically insignificant trends towards lowering of these monthly sick leave figures in Kalmar (reduction of sick leave for F99 p = 0.062, and for diagnoses M99, p = 0.098) while there were no such trends in Jönköping (Table 2).

As data was lacking from Högsby regarding the educational activities that were attended we had very low statistical power to detect relationships between attended educational activities and potential changes in sick leave. We found no such correlations when testing linear regression (data not shown). There were also only 4 subjects who handed in filled-out questionnaires on participation rate from Färjestaden in our data base. It could be noted that it was only in Färjestaden that there was no numerical drop in sick leave in F43 when comparing 2019 with 2017 (Table 1) and in this center there were also no dedicated educational activities for physicians (see supplementary file).

## Discussion

To the best of our knowledge the design of the trial presented herein, with ACT-based CBT directed towards the staff rather than to the patients, has not been performed earlier. We tested the hypothesis that a more accepting and hence potentially productive attitude towards disabling health issues could be conveyed from the staff that had been educated by ACT and found a 21% reduction of sick leaves for any diagnosis during the second year of the intervention. We also hypothesized that health issues under ICD-10 F43 were the ones that might particularly benefit from ACT as part of the main aim of the trial. Secondarily we also studied sick leave for musculoskeletal issues and the participants could attend extra lectures on how to handle patients with pain problems. While sick leave prescriptions for the diagnosis F43 fell by 37% in 2019 in Kalmar, compared to 2017, there was an unsignificant trend for sick leave reduction due to musculoskeletal health problems. This is in line with the fact that musculoskeletal disease might require sick leave for reasons that cannot very readily be affected by the educational activities. Indeed, it is well known that there were, even before the Corona-pandemic that started in early 2020, long waiting lists for orthopedic surgery of both coxarthrosis and gonarthrosis in Kalmar.

The main issue for the health care resources, however, was that the reduction in sick leave for F43 was not explained by change of the specific diagnosis to some other code, as there were also trends of a lowered sick leave in M99 and F99. In particular total sick leave was reduced in 2019 compared to 2017. The ACT based educations of the staff were mainly aimed to deal with problem areas such as pain and psychosocial and psychiatric diseases, but we acknowledge that ACT has a potential to be helpful in coping with many kinds of general distress [21–25].

We acknowledge that we cannot prove that it was the actual interventions in Kalmar that led to better health of the patients. We merely showed that doctors did not prescribe as many or lengthy sick leave certificates. However, since there was no backlash of the reduction of sick leave during the second year, we believe that it is unlikely that ACT hindered continuing rehabilitation of the affected patients. In line with this we demonstrated a reduction of sick leave of 3–12 months for any diagnosis when comparing the period at the start of the trial with the last month of the study period.

To the best of our knowledge, there have been no other trials of how to reduce sick leave in Scandinavia that has reached similar magnitudes of reductions before. The cost of having educational activities for the staff at the primary health care centers whom on average attended 5 educational activities/year is apparently quite minute compared to the societal savings encountered in reduced sick leave costs. The cost reduction corresponding to 21% less total sick leaves could be allocated to for example shorten the waiting list for surgery of arthrosis, or some other therapeutic measure with clear effects on the well-being of patients in primary health care.

### Limitations

We acknowledge that we had no data for sick leave after two years, as this was not part of the trial design. In retrospect, based on the clinically relevant effects, we discussed addition of adding data from early months of the year 2020. However, since the Corona pandemic struck Sweden in the early spring of 2020, we assumed that such data would be very hard to interpret. Indeed, the Swedish government acutely changed sick leave regulations following this pandemic allowing patients to stay home longer periods without a sick leave prescription.

The figures on attendance of the educational activities had significant loss of data. There were no data at all from Högsby and we had only 4 respondents in Färjestaden. In these two health care centers Kadowaki did not give dedicated education to the physicians. This might have been linked with the fact that Färjestaden was the only center in which there was not a numerical drop in F43 sick leave after two years. In Högsby, on the other hand, Kadowaki had several meetings with the nurses and there was a strong numerical decrease in sick leave in this health care center as seen in Table 1. However, the lack of reliable data from all participating centers gave poor statistical power to assess potential relationships between extent of education and related reduction of sick leave prescriptions. Thus, we cannot rule out that any large-scale project with other kinds of educations of the clinical staff to affect sick leave prescription could have had a similar effect. However, since we did find significant effects on sick leave for ICD-10 F43 this was in line with the hypothesized effects of ACT-based education of the staff.

The fact that Eksjö had a particularly large sick leave prescription, could theoretically have afflicted the figures. However, removal of Eksjö from the statistics in Region Jönköping did not accomplish a diminishing trend in any of the diagnoses that were part of the trial (post hoc analysis, data not shown). In this respect it should also be noted that we did not chose control health care centers based on similar sick leave frequency, but rather on the fact that Jönköping is the region geographically closest to Kalmar and that no ACT-based educations were given in this region during this time period.

All our investigations were undertaken in South East Sweden and it is possible that a similar effect on reduction of sick leave prescription would not have been achieved elsewhere. It is also possible that the results were linked with enthusiastic educational activities and organization from doctor Kadowaki, the results of which might not be equally powerful when applied by others.

The major limitation of our study presented here was the non-randomized design. The reduction in sick leave could theoretically have been due to chance, or by other factors that were not identified by the data at hand, in Kalmar. We thus acknowledge that the report herein could be seen as a pilot trial, merely allowing the findings to serve as a basis for randomized trials on the same theme in which the potential change in attitudes of the staff could be given more systematic attention.

## Conclusion

Our findings of a clinically relevant 21% reduction of total sick leaves/month after two years in 6 health care centers in which ACT based education of physicians, nurses and therapists was given encourages further investigations in a regular randomized trial setting using the data presented herein for calculation of statistical power.

## Supplementary Information

**Additional file 1: Supporting File 1.**

## Data Availability

The original data will be sent to other scientists on reasonable request to the corresponding author.

## Abbreviations

ACT: Acceptance and Commitment Therapy
CBT: Cognitive behavioral therapy
GP: General practitioners
ICD: International Statistical Classification of Diseases and Related Health Problems
