# Supplementary material for: Education of the primary health care staff based on acceptance and commitment therapy is associated with reduced sick leave in a prospective controlled trial
Source: BMC Fam Pract. 2021 Sep 8;22:179. doi: 10.1186/s12875-021-01526-5 (PMC8428055; doi:10.1186/s12875-021-01526-5)
Supplement: Supplementary file 1 — Additional file 1: Supporting File 1. [file 12875_2021_1526_MOESM1_ESM.docx]

**Supporting File 1.**

**Methods, addendum:**

In more detail the health care centers received the following educational activities in Kalmar:

In Färjestaden all personnel were educated at the same occasion in the beginning of the trial when the questionnaires were handed out. There was no particular separate education of physicians. Some physical therapists participated in education-activities at other locations close by, also led by Kadowaki. In Ljungbyholm the physicians participated in group-based educational activities with other health care centers. The remaining personnel received ACT-based education in large groups during an hour. Nurses also received separate educational activities and the rehabilitation coordinator was separately guided in some particularly cumbersome patient cases. In Högsby there were many physicians who worked for short periods of times (i.e. “rental-doctors”) that did not belong to the permanent staff, and they did not participate in the educations. However, the nurses and rehabilitation coordinators were educated for about 6-8 times/year and they conveyed information on how to aim to cope with sick leave requests at the health care center to the physicians. On some occasions the physical- and occupational therapists also participated in the meetings with Åsa Kadowaki. In Emmaboda the physicians mostly participated in coordinated educations together with other physicians in the city of Kalmar. The nurses, physical- and occupational therapists had separate educations. During late 2019 Åsa Kadowaki also held meetings in large groups for all personnel in which focus was on that rest is less effective than activities in the rehabilitation process in accordance with ACT. Stora Trädgårdsgatan and Esplanaden are both located in the city of Västervik. Here the physicians mostly participated in coordinated educations with other physicians in the city of Kalmar but Åsa Kadowaki also participated under some day-long local educational activities. Nurses, physical- and occupational therapists had separate ACT-educations. Åsa Kadowaki also gave advice in extra complicated cases in group sessions.
